# Is Shorter Better in Oncology Patients, Too? A Retrospective Cohort Study of Short- Versus Long-Course Antibiotic Therapy for Uncomplicated Infections in Solid Tumor Patients Receiving Care in Ambulatory Oncology Clinics

**DOI:** 10.1093/ofid/ofaf505

**Published:** 2025-08-21

**Authors:** Christen J Arena, Beisan El-Tatari, Kristen Lovric, Sage B Greenlee, Rachel M Kenney, Shirish M Gadgeel, Katelyn Patterson, Anita B Shallal, George J Alangaden, Susan L Davis, Michael P Veve

**Affiliations:** Department of Pharmacy, Henry Ford Hospital, Detroit, Michigan, USA; Department of Pharmacy Practice, Eugene Applebaum College of Pharmacy and Health Sciences, Wayne State University, Detroit, Michigan, USA; Department of Pharmacy, Henry Ford Hospital, Detroit, Michigan, USA; Department of Pharmacy Practice, Eugene Applebaum College of Pharmacy and Health Sciences, Wayne State University, Detroit, Michigan, USA; Department of Pharmacy, Henry Ford Hospital, Detroit, Michigan, USA; Department of Pharmacy Practice, Eugene Applebaum College of Pharmacy and Health Sciences, Wayne State University, Detroit, Michigan, USA; Department of Pharmacy, Henry Ford Macomb Hospital, Clinton Township, Michigan, USA; Department of Pharmacy, Henry Ford Hospital, Detroit, Michigan, USA; Division of Hematology and Oncology, Henry Ford Hospital, Detroit, Michigan, USA; Department of Internal Medicine, Henry Ford Hospital, Detroit, Michigan, USA; Department of Pharmacy, Henry Ford Cancer Institute, Detroit, Michigan, USA; Division of Infectious Diseases, Henry Ford Hospital, Detroit, Michigan, USA; Division of Infectious Diseases, Henry Ford Hospital, Detroit, Michigan, USA; Department of Pharmacy Practice, Eugene Applebaum College of Pharmacy and Health Sciences, Wayne State University, Detroit, Michigan, USA; Department of Pharmacy, Henry Ford Hospital, Detroit, Michigan, USA; Department of Pharmacy Practice, Eugene Applebaum College of Pharmacy and Health Sciences, Wayne State University, Detroit, Michigan, USA

**Keywords:** antimicrobial stewardship, ambulatory stewardship, outpatient stewardship, immunocompromised, Oncology, solid tumor, short vs. long antibiotic durations

## Abstract

This retrospective cohort study evaluated short- versus long-course antibiotics for uncomplicated infections in ambulatory solid tumor patients. Among 303 patients, outcomes were similar between groups, including infection recurrence, treatment delays, and adverse events. Short-course therapy was not associated with worse outcomes, suggesting it may be a viable alternative.

## BACKGROUND

Patients with cancer represent populations where progressive antimicrobial stewardship practices are needed [1–3]. Current data suggest antimicrobial stewardship interventions designed and evaluated in general populations can be safely extrapolated to patients with cancer [4, 5]; however, implementing these practices in cancer care is challenging due to lack of specific data, infection diagnostic uncertainties, clinician perceptions, complexities and concerns in immunocompromised population care, and increased risk of infection and death due to multi-drug resistant organisms [1–6]. Nonetheless, literature suggests that patients with cancer could and should be evaluated for meaningful stewardship interventions, such as short-course antibiotic durations [1].

Optimizing antibiotic duration is a core antimicrobial stewardship strategy that reduces adverse drug events and resistance [7]. While short-course antibiotics have proven effective in many trials, cancer patients in ambulatory settings are often underrepresented [1, 2, 4, 5]. This gap is critical as outpatient oncology care grows and stewardship remains limited [1, 2, 8, 9]. Prolonged antibiotic use increases harm, particularly in immunocompromised patients, who face higher risks of adverse drug events (ADEs), *Clostridioides difficile* infection (CDI), and resistance [1, 4, 5, 10, 11]. Despite national guidelines favoring long-course therapy, emerging evidence suggests shorter durations may be equally effective and safer [1, 12–16]. This study evaluates short- versus long-course antibiotics for uncomplicated infections in ambulatory solid tumor patients.

## METHODS

### Study Design

This was an IRB-approved, non-inferiority, retrospective cohort of patients who received short-course and long-course antibiotic therapy from ambulatory oncology clinics within Henry Ford Health, located throughout southeast Michigan, from 1 January 2014 to 31 December 2024.

Patients ≥18 years old with an active solid tumor diagnosis who received cancer treatment ≤3 months of initial antibiotic prescription, and received an oral antibiotic for a urinary tract infection (UTI), lower respiratory tract infection (LRTI), or an acute bacterial skin–skin structure infection (ABSSSI) in an ambulatory oncology clinic were included. These infection types were selected for their lower risk of complications and high outpatient prevalence [1, 2]. Diagnostic criteria are detailed in Supplementary Material 1. Patients were categorized to short- and long-course antibiotic therapy per infection type based on best practice and National Comprehensive Cancer Network guidance (Table 1) [12]. Exclusions included neutropenia (ie, absolute neutrophil count <500 cells/μL), antibiotic prophylaxis, multiple or complicated infections, upper respiratory tract infections, checkpoint inhibitor-pneumonitis, and hematologic malignancies to reduce population heterogeneity.

**Table 1.** Short- and Long-Course Antibiotic Therapy Definitions Based on Recommended Evidence-Based Medicine and National Comprehensive Cancer Network (NCCN) Guidance

Infection Type	Short-Course Therapy Recommended—Evidence-Based [13–16]	Suggested Minimum Duration of Therapy For Documented Infection [12]	Study Definition Short-Course	Study Definition Long-Course
ABSSSI (ie, non-purulent and purulent cellulitis)	5–6 d	5–14 d	≤5 d	>5 d
LRTI (ie, CAP and COPD exacerbation)	3–5 d	5–14 d	≤5 d	>5 d
UTI				
Complicated cystitis	7 d	7 d	≤6 d^a^	>6 d^a^
Pyelonephritis	7 d	10–14 d	≤7 d	>7 d

Abbreviations: ABSSSI, acute bacterial skin and skin structure infection; LRTI, lower respiratory tract infection; CAP, community-acquired pneumonia; COPD, chronic obstructive pulmonary disease; UTI, urinary tract infection.

^a^A 6-d threshold to define short-duration therapy for complicated cystitis was chosen to reflect common prescribing patterns at HFH, where clinicians often do not distinguish between uncomplicated and complicated lower UTI cases.

The primary endpoint was a 30-day composite of (1) probable or proven infection-related recurrence or (2) need for additional antibiotic therapy post end of therapy from index encounter. Probable recurrence involved symptom return with a new antibiotic prescription of the infection responsible for the index encounter after symptom resolution; proven recurrence included culture-confirmed infection after index symptom resolution. “Required additional antibiotic therapy” was defined as receipt of an additional antibiotic prescription that was prescribed within 30 days of the index antibiotic prescription. Secondary 30- and 90-day outcomes included cancer treatment delays due to infection and antibiotic-associated ADEs as documented in the electronic health record (EHR).

### Patient Data

Patient cohorts were identified through a standardized EHR screening process. International Classification of Diseases, Tenth Revision codes related to diagnosis name and an outpatient encounter were used to identify patient encounters where antimicrobials were ordered (Supplementary Material 1). ICD-9 codes were not included due to inconsistencies in documentation and system transitions during the early years of the study period. Patient data were then manually screened for inclusion; only outpatient encounters where an oral antibiotic was prescribed were included. Patients were excluded primarily due to prophylactic antibiotic use, multiple concurrent infections, or infections not meeting predefined criteria for uncomplicated cases. Screening data were extracted using Microsoft SQL Server Management Studio (Microsoft Corp., Redmond, WA).

### Statistical Analyses

The non-inferiority sample size calculation was based on an alpha of 2.5%, power of 80%, percentage success in the 2 groups of 80%, and a non-inferiority limit of 10%. A total of 504 patients were needed to be 80% sure that the upper limit of a one-sided 97.5% confidence interval would exclude a difference in favor of the long-course group of >10% [2].

Descriptive statistics (*n*, [%], median [IQR]) were used to summarize patient cohorts. Bivariate analyses included the Mann–Whitney *U* test for continuous variables and Chi-squared or Fisher's exact tests for categorical variables. Variables with *P* < .20 were entered into a multivariable logistic regression using backward stepwise selection, with collinearity assessed and variable inclusion guided by effect size. A 10:1 event-to-variable ratio was maintained, and model fit was evaluated using the Hosmer–Lemeshow test. Analyses were conducted using SPSS v26.0 (IBM, Armonk, NY).

## RESULTS

### Patient Characteristics

Three-hundred and three patients were included; 92 (30%) and 211 (70%) received short- and long-course antibiotic therapy, respectively. Baseline demographics for short- and long-course groups are listed in Table 2a. Most patients had a primary breast cancer diagnosis (115, 40%) followed by lung cancer (62, 21%). One-hundred ninety-eight (66%) patients had metastatic disease at the time of the encounter. Two-hundred fifty-six (84%) patients had a documented ECOG score, with a median (IQR) score of 1 (1–2) in the short-course and 1 (0–1) in the long-course group (*P* = .060).

**Table 2.** Patient Demographics, Outcomes, Treatment Characteristics, and Multivariable Regression of Patients With Cancer Who Received Care at an Ambulatory Cancer Clinic

(a) Variable, *n* (%) or Median (Interquartile Range)	Short-Course Antibiotic Therapy *n* = 92	Long-Course Antibiotic Therapy *n* = 211	*P* Value
Age (y)	65 (57–72)	65 (55–73)	.636
Charlson comorbidity index	9 (6–10)	8 (6–10)	.208
Sex assigned at birth, Woman	63 (69%)	155 (74%)	.375
Race, white	68 (74%)	150 (71%)	.615
Ethnicity, not Hispanic or Latinx/o	84 (91%)	197 (93%)	.525
Antibiotic allergy	37 (40%)	81 (38%)	.764
Primary cancer			
Breast	28 (30%)	87 (41%)	.075
Lung	25 (27%)	37 (18%)	.056
Other	39 (42%)	87 (41%)	.851
Metastatic cancer	64 (70%)	134 (64%)	.308
Infection type			
Cystitis/lower UTI	36 (39%)	60 (28%)	.066
Upper UTI	6 (7%)	0	<.001
COPD exacerbation	7 (8%)	4 (2%)	.021
CAP	22 (24%)	17 (8%)	<.001
ABSSSI	21 (23%)	130 (62%)	<.001
Microbiological culture collected	33 (36%)	50 (24%)	.031
Risk factors for MRSA and *P. aeruginosa*			
History of MDRO	0	3 (1%)	.556
Hospitalization within 90-d	27 (29%)	63 (30%)	1.000
Parenteral antibiotic use within 90-d	11 (12%)	29 (14%)	.673
Treatment for same infection within 90-d	17 (19%)	37 (18%)	.844
30- and 90-d patient outcomes			
Probable or proven infection-related recurrence or required additional antibiotic therapy			
30-d	11 (12%)	34 (16%)	.349
90-d	20 (22%)	39 (19%)	.510
Infection-related hospital admission			
30-d	4 (4%)	16 (8%)	.297
90-d	7 (8%)	14 (7%)	.759
Antibiotic-associated ADE			
30-d	1 (1%)	2 (1%)	1.000
90-d	0	1 (1%)	1.000

(b) Variables Associated With 30-d Infection Recurrence/Re-Use of Antibiotics			
Variable	UnAdjOR (95% CI)	*P* Value	AdjOR (95% CI)
Short-course antibiotic therapy	1.41 (0.68–2.93)	.34	0.48 (0.15–1.50)
Antibiotic allergy	1.79 (0.95–3.39)	.07	0.67 (0.25–1.83)
Lung cancer diagnosis	1.73 (0.85–3.55)	.13	2.83 (1.10–7.26)
Sex assigned at birth, woman	0.95 (0.47–1.92)	.89	Not Tested

Abbreviations: UTI, urinary tract infections; COPD, chronic obstructive pulmonary disease; CAP, community-acquired pneumonia; ABSSSI, acute bacterial skin and skin structure infection; MDRO, multidrug-resistant organism; ADE, adverse drug event; UnAdjOR, unadjusted odds ratio; CI, confidence interval; AdjOR, adjusted odds ratio.

### Infection and Treatment Characteristics

The most common infection types were ABSSSIs (151, 50%), followed by UTIs (102, 34%), and LRTIs (50, 17%). Short-course therapy was more common in patients with UTIs (42, 46%) and LRTIs (29, 32%). The median (IQR) duration antibiotic therapy for the short- and long-course groups were 5 (5–5) and 7 (7–10) days, respectively (Supplementary Material 2). Cultures were obtained in 36% of short-course and 24% of long-course patients (*P* = .029).

### Short-Course Antibiotic Therapy Was Not Associated With 30-Day Infection Recurrence/Re-Use of Antibiotics

A formal non-inferiority analysis was not performed due to infection heterogeneity. Based on bivariate results and clinical rationale, short-course therapy, lung cancer, and antibiotic allergy were included in the multivariable model (Table 2b). Other variables were excluded from the model due to unmet statistical criteria or due to co-variability. After adjustment, short-course therapy was not independently associated with 30-day probable of proven infection-related recurrence or additional antibiotic therapy (adjOR, 0.478; 95% CI 0.152–1.501).

### Secondary Outcomes

There were no statistical differences in 30- or 90-day secondary outcomes between the short- and long-course therapy cohorts (Table 2a). About 12% of the short- and 15% of the long-course had a delay in cancer treatment due to infection (*P* = .526). Of the 30-day ADEs, one short-course patient experienced gastrointestinal upset with ciprofloxacin use even when the antibiotic was taken with food and was switched to sulfamethoxazole-trimethoprim to complete the treatment. Of the 2 long-course patients who experienced an antibiotic-associated ADE, one patient experienced gastrointestinal upset after 6 doses of nitrofurantoin and the other patient developed CDI. Ninety-day patient outcomes also revealed no differences between the cohorts.

## DISCUSSION

Short-course antibiotic therapy was not associated with 30-day infection recurrence or additional antibiotic use in ambulatory solid tumor patients with uncomplicated infections. No differences were observed in secondary 30- or 90-day outcomes.

Although short course prescribing was infrequent, these findings suggest oncology specialists are beginning to adopt a “shorter is better” approach, even without solid tumor-specific trial data. This study did not show fewer ADEs with prolonged therapy. However, prior research has consistently demonstrated this benefit with shorter antibiotic durations [1, 4, 10, 17–20].

Although solid tumors are more common than hematologic malignancies, infections in this population remain understudied, especially regarding short-course antibiotic use in ambulatory settings [21]. Chew and colleagues assessed antibiotic appropriateness in 411 solid tumor patients, with 81% receiving durations aligned with NCCN guidelines [22]. These results are similar to the present study, but they did not define short-course therapy or compare outcomes by duration.

While antimicrobial stewardship efforts in cancer patients have shown success, most studies focus on hospitalized patients with hematologic malignancies [1, 23–27]. While not directly applicable, studies in hospitalized immunocompromised patients show no difference in outcomes between short- and long-course antibiotic therapy [24–27]. Since most data involve neutropenic or critically ill patients, these findings may reasonably extend to cancer populations with less severe infections [2, 5].

While this study offers valuable insights into antibiotic use for ambulatory solid tumor patients, several limitations exist. The median 7-day duration in the long-course group reflects relatively progressive prescribing, limiting the difference between groups and making harm detection challenging. Heterogeneity in cancer types and infection distribution may have introduced confounding by indication, illness severity, or chemotherapy-related risks, despite efforts to minimize variability through strict criteria and multivariable modeling. Reliance on ICD-10 codes and exclusion of ICD-9 may have reduced sample size. This study was underpowered to establish non-inferiority and may be subject to Type II error. Use of a composite outcome across varied infections, potential colonization, and reliance on EHR documentations also limit interpretation. Additionally, evolving prescribing practices over the study period and institutional variability may affect generalizability. Future studies should focus on single infection types for continuity of outcome assessment and use robust observational or randomized designs [28].

## CONCLUSIONS

This study found no significant difference in outcomes between short- and long-course antibiotics for uncomplicated infections in ambulatory solid tumor patients. These findings suggest safety and effectiveness of short-course therapy in select immunocompromised patients, with potential benefits including reduced resistance and adverse events, improved adherence, and fewer treatment delays. Future research should focus on implementing stewardship strategies to optimize short-course use in this population [1, 2].

## Supplementary Material

ofaf505_Supplementary_Data

## Supplementary Data

Supplementary materials are available at *Open Forum Infectious Diseases* online. Consisting of data provided by the authors to benefit the reader, the posted materials are not copyedited and are the sole responsibility of the authors, so questions or comments should be addressed to the corresponding author.
