# Supplementary material for: Is Shorter Better in Oncology Patients, Too? A Retrospective Cohort Study of Short- Versus Long-Course Antibiotic Therapy for Uncomplicated Infections in Solid Tumor Patients Receiving Care in Ambulatory Oncology Clinics
Source: Open Forum Infect Dis. 2025 Aug 21;12(9):ofaf505. doi: 10.1093/ofid/ofaf505 (PMC12405994; doi:10.1093/ofid/ofaf505)
Supplement: ofaf505_Supplementary_Data [file ofaf505_supplementary_data.docx]

**Supplemental Material**

**Supplement 1**. International Classification of Diseases, Tenth Revision codes and diseases state definitions

| **Disease State** | **Definition** |
| --- | --- |
| **Urinary Tract Infections (UTIs)** | |
| Complicated cystitis (lower tract infection)  ICD10 Code: ‘N30.xx’  Dx name like: ‘cystitis’, ‘urinary tract infection’ | Urinary or constitutional symptoms confirmed by the presence of bacteriuria in patients without symptoms of upper tract infection (flank pain) who do not meet criteria for uncomplicated cystitis (i.e., male patients, immunosuppressed patients, patients with urinary catheters, etc.). |
| Complicated upper urinary tract infection  ICD10 Code: 'N10.xx’,  Dx name like: ‘UTI’, ‘urinary tract infection’, ‘pyelonephritis’ | Pyelonephritis (flank pain and/or fever confirmed by bacteriuria) or other urinary infection involving the ureters or kidneys. |
| **Lower Respiratory Tract Infections (LRTIs)** | |
| COPD Exacerbation  ICD10 Code: ‘J44.xx’  Dx name like: ‘COPD exacerbation’ | Increased sputum purulence PLUS increased dyspnea and/or sputum volume. |
| Community-acquired pneumonia (CAP)  ICD10 Code: ‘J18.xx’  Dx name like: ‘pneumonia’ | *Uncomplicated CAP*: not meeting criteria for complicated CAP.  *Complicated CAP*: moderate immune compromise, structural lung disease, respiratory culture with Staphylococcus aureus or a non-fermenting gram-negative bacilli (*Pseudomonas* spp.), or moderate to severe COPD.  *Severe CAP*: either 1 major criterion or ³3 minor criteria  -Major criteria:   - Septic shock w/ need for vasopressors - Respiratory failure requiring mechanical ventilation   -Minor criteria:   - Respiratory rate ³30 breaths/min - PaO2/FiO2 ratio £250 - Multilobar infiltrates - Confusion/disorientation - Uremia (BUN level ³20 mg/dL) - Leukopenia due to infection (WBC <4,000 cells/mcgL) - Thrombocytopenia (platelet count <100,000 mcg/L) - Hypothermia (core temperature <36°C) - Hypotension requiring aggressive fluid resuscitation |
| **Acute Bacterial Skin and Skin Structure Infections (ABSSSIs)** | |
| Cellulitis (non-purulent and purulent)  ICD10 Code: ‘L03.xx’  Dx name like: ‘cellulitis’, ‘abscess’ | Microbial invasion of the layers of the skin and underlying soft tissues. |

**Supplement 2.** Antibiotic duration of therapy distributions for lower respiratory tract infections (A), urinary tract infections (B), and acute bacterial skin and skin structure infections (C)
